# Investing Time and Resources for Work–Life Balance: The Effect on Talent Retention

**DOI:** 10.3390/ijerph17061920

**Published:** 2020-03-16

**Authors:** José-Luis Rodríguez-Sánchez, Thais González-Torres, Antonio Montero-Navarro, Rocío Gallego-Losada

**Affiliations:** Facultad de Ciencias Jurídicas y Sociales, Universidad Rey Juan Carlos, Paseo de los Artilleros, s/n 28032 Madrid, Spain; thais.gonzalez@urjc.es (T.G.-T.); antonio.montero@urjc.es (A.M.-N.); rocio.gallego@urjc.es (R.G.-L.)

**Keywords:** talent, retention, valuable human resources, work–life balance, flexibility, training

## Abstract

The study of work–life balance has undergone significant development in recent years as a result of changes in society and the growing importance of human resources (HR) for companies. Taking into account that human capital represents a critical success factor for businesses, the current context requires the development and implementation of HR management strategies aimed at attracting and retaining the most talented workers in order to obtain the expected results. The objective of this paper is to present an integrated model of work–life balance strategies, including the impacts of the different policies and practices on the retention of talented HR, which can be a basis for further academic developments on this subject, as well as a roadmap for managers. Hence, we will analyze a case study carried out in a multinational company—a leader in the technology and tourism sectors, and importantly dependent on valuable human capital, for which the HR strategy aims to improve the performance of the firm in the medium and long term through analysis, planning, and flexibility.

## 1. Introduction

In the current business and social environments, firms are increasingly aware of the importance of employees with excellent abilities—also called key employees—for achieving success [1]. These talented workers with high potential are particularly valuable to organizations, so they become a scarce resource to be attracted and retained [2,3]. In this regard, the study of Juarez-Tarraga, Santandreu-Mascarell, and Marin-Garcia [4] reveals that among the challenges most frequently mentioned by human resources (HR) professionals are retaining the best employees, talent management, and employee engagement. Moreover, concern about talent management is also shared by CEOs.

This research also falls within the framework of strategic human resource management, which is based on the resource-based view of the firm, thus highlighting the uniqueness of the workers’ competences for organizational success [5], as well as individual commitment, skills, and knowledge [6].

Traditionally, factors such as security and salary were critical to attract and retain valuable HR. However, with a few exceptions, in the current European context the employee salary cannot be considered a key determinant, since it largely depends on the current labor legislation [7,8]. In addition, globalization has contributed to increasing the competitiveness of the labor market, making it more difficult for companies to attract and retain the most outstanding and qualified human resources [2]. In this way, forms of remuneration based on individual performance are considered critical sources of employee engagement, given that they contribute to aligning organization and staff interests, improving productivity and the firm’s key indicators [7].

In addition to traditional compensation, new generations joining the labor market are more interested in jobs that respect their spare time, offer them the possibility to practice sports, improve their training, or give them a chance to increase social welfare [9]. Nowadays, workers look for incentive policies and benefits that allow them to be happier, beyond simply caring for their salary. In other words, societies are increasingly concerned about work–life balance, personal well-being, and job satisfaction. 

These business and social changes have led organizations to modify their vision and strategy regarding HR management, and therefore to find formulas to attract and engage valuable HR. Among the main factors driving talent retention goals are having faith in the organization, a sense of belonging, and day-to-day motivation [10]. Accordingly, organizations must be aware of the relevance of factors such as non-monetary benefits in order to avoid the loss of motivation of talented employees, absenteeism, or voluntary abandonment [10].

In this regard, the development of work flexibility policies is beginning to have positive results for both workers and companies [11]. For the employees, flexibility practices can reduce stress, fatigue, and conflict. For the firm, it implies higher levels of commitment, which improves productivity rates and reduces recruitment costs resulting from lower staff turnover. Hence, the implementation of work–life balance policies represents a potential retention strategy for valuable and talented HR. However, in practice, in many firms there is still a lack of application of such strategies [6,12]. 

In this context, the main objective of this article is to analyze the determining factors for the retention of valuable HR through work–life balance strategies. In order to achieve this objective, a case study of a multinational company is developed. It is focused on discovering the reasons for the current talent crisis and analyzing the impact of work–life balance policies on the attraction and retention of valuable HR. The analysis of the information gathered will enable us to know how investing time and resources in managing work–life balance helps to attract and retain valuable HR. 

Therefore, we aim to identify the specific impacts that the main work–life balance policies have on HR retention. This result could, in turn, become the basis for further academic developments, analyzing the most suitable policies for different kinds of environments and organizations, as well as could serve as a roadmap for practitioners to reinforce talent retention.

The article is organized as follows. The next section develops the theoretical foundations of the subsequent case study, establishing the determinant factors driving work–life balance policies and retention of talented HR through these strategies. The third section addresses the methodology used. In the fourth section, the results obtained are presented and analyzed. In the last section, the main conclusions, implications, and limitations of the article, as well as the future lines of research, are presented. 

## 2. Theoretical Framework

### 2.1. Societal Changes Driving Work–Life Balance Policies

Firms no longer conceive HR as resources whose cost should be minimized, but rather as strategic assets. In this regard, economic and social changes have led companies to become more involved in the problems of HR at work and also in their personal lives.

Globalization and competiveness are critical issues when addressing HR management. The emerging markets are nurturing their own growth through young HR, many of them coming from other countries [13]. As a result, the labor market has become global, leading to an interdependent economy [10]. Organizations must be aware of this context in order to achieve and maintain a sustainable competitive advantage. In this context, companies find themselves in a costly and complex situation to recruit and retain the best talent, whatever their origins or culture might be [11]. From a general point of view, the most valuable HR are those who are technological experts, operationally agile, and have a global mentality that works locally [14].

In addition to economic trends, social changes are also occurring. Almost two decades ago, experts in HR and organizational behavior began to be concerned about the aging of baby boomers and the decrease in birth rates, which would give rise to a talent crisis in companies [13].

In Europe, we are experiencing a “demographic winter”, which anticipates an immediate problem for the recruitment and retention of talent [15]. Due to the aging of the population and the decrease in birth rates, it is harder to find talent. Companies are forced to squeeze their older HR because of the lack of a younger workforce [16].

Furthermore, in the past 10 years, the Y generation, also known as millenials, have joined the labor market. Currently, they compose the HR segment that is growing the most in the majority of countries. The millenials have behaviors, values, and attitudes that are very different from those of the preceding generations [2]. As an example, in contrast to the baby boomers, for these employees, family is more important than work [13]. Additionally, feeling committed to an organization is a key factor for them [17]. Consequently, they are more interested in jobs that respect their free time and offer them opportunities to practice sports, improve their training, or dedicate themselves to improving social well-being, above and beyond the salary [11].

Finally, in competitive environments such as the current one, there is a continuous demand for HR to give their best. Long work shifts and rigid schedules can decrease the predisposition to work and reduce employees’ creative and innovative capacities [18]. Furthermore, difficulties in finding a balance between work and personal life lead to stress and emotional fatigue, contributing to inefficiencies and mistakes [19]. These elements have become an important source of HR turnover [20].

### 2.2. Work–Life Balance Policies and Talent Management

In the highly competitive environment in which businesses operate, it is essential to rely on the most talented employees, not only to develop the activities of the firm, but also to survive and succeed [2]. Talent requires specific management, since HR are critical for business survival in the current situation of complexity and uncertainty. The notion of talent is usually defined as “excellent abilities”, however it also refers to key employees—those individuals with high potential who are particularly valuable for an organization [1]. Accordingly, within the framework of strategic human resource management (HRM), talent management practices are focused on the systematic attraction, identification, development, engagement and retention, and deployment of talents [21]. All this has led many organizations to use talent managers in HR departments.

Corporate social responsibility (CSR) is such a critical tool for talent management that some authors advocate a co-created model, where CSR and HRM practices are designed together. On the one hand, CSR policies are potential instruments to attract talented individuals to organizations given the higher levels of attractiveness of socially responsible firms [22]. On the other hand, CSR practices of firms can directly impact the positive relationships between a company and the workforce. Thus, socially responsible firms will facilitate employee identification with them, which in turn will influence employees’ commitment and retention [23]. From a general point of view, CSR as a long-term strategy is associated with firm-level outcomes, such as firm performance and shareholders’ benefits [24]. Talent management practices also result in different organizational outcomes, such as company attractiveness, the achievement of business goals, and corporate profit. Moreover, these practices also have a positive impact on human resource outcomes, such as job satisfaction, motivation, commitment, and trust in leaders [25].

It should be noted that substituting talented and valuable HR has high associated costs, while investing in HR is much less expensive than replacing them [16]. The direct costs associated with the loss of these types of workers include the costs of substituting them, the inactivity in their job position, the period of recruitment and hiring, and the training of the new employee [26]. Some authors estimate these costs at between 1.5 and 2.5 times the annual salary of the lost worker [27]. Furthermore, there are also indirect costs, such as interruption of work, loss of organizational memory, and decreased strategic knowledge and productivity [10]. In this context, firms must be aware of the relevance of retaining valuable and talented workers. 

Although traditionally the salary as the main part of the reward for a worker, some other compensation schemes, such as the use of financial instruments, have been frequently used to increase the involvement of executives, who can be considered critical employees. In the case of equity incentives (shares and stock options), firms must be aware of their size, growth opportunities, and proxies for monitoring costs in order to design an optimal portfolio of incentives [28]. The use of promises of receiving fixed sums in the future, such as pensions or deferred compensations, frequently called inside debt [29], is based on the sensitivity of managers to the firm’s bankruptcy risk, as well as to the firm’s value in the event of bankruptcy. In this context, a credit crisis can result in an increase in executive conservatism [29]. These kinds of reward schemes can also be used for other talented employees [30,31].

In addition to the widespread consideration of talent, significant demographic, economic, and cultural changes are now taking place, such as rising numbers of women in the labor force, transformation of the family structure, an ageing population, and technological advances enabling near constant contact with the workplace. These factors have led employees to become increasingly concerned with harmonizing demands between work and home. Karatepe and Udalug [32] observe that employees that have difficulty spending time with their families or finding time for their social relationships are more likely to be being emotionally tired. This fatigue, in turn, has an impact on their dissatisfaction with their job and an influence on their intention to leave the organization [13,27]. In addition, overworked employees often create a climate of discontent, leading to decreased job performance [33]. In this context, organizations are forced to adopt work practices and compensation policies intended to balance the worker´s employment-related and personal responsibilities [20,34].

Optimizing the reward system is very important for retaining the best workers without falling into an increase in payroll [35]. Among the critical factors of the reward systems are security, location, relationships, recognition, contribution, salary, flexibility, learning, responsibility, and innovation [36]. In this vein, work–life balance practices represent an HRM strategy that organizations can use to address a great range of the previous factors [6,33]. These policies represent the organizational support for dependent care, flexible work options, and family or personal leave [27,37]. This includes family leave programs, on-site childcare, assistance with childcare and eldercare services, flexible work hours, telework, and job sharing. By offering these alternatives, firms should not only attract individuals to an organization but also improve attitudes and behaviors within it and reduce work–life conflict among employees, leading to higher levels of retention [10,34]. 

The implementation of work–life balance measures is a real challenge, since it can facilitate career development and simultaneously fulfilment and satisfaction with personal lives [38]. Employees whose professional careers are well managed and that feel happy in their work are more likely to stay in the organization [39]. However, the complex nature of human capital makes it difficult to develop a standard package of work–life balance measures [40]. Nevertheless, higher levels of satisfaction and retention as a result of work–life balance practices represents a potential source of competitive advantage for firms, both in terms of productivity and differentiation from competitors [10,18,33,41].

In the current social context, we cannot conceive of the company and the family as autonomous entities, rather as two interdependent entities that may have a positive influence on each other. Accordingly, it is necessary for firms to adopt a proactive approach in order to implement work–life balance measures [39].

## 3. Methodology

According to Merriam [42], the case study methodology, within the qualitative research, enables a deeper understanding of a specific social phenomenon. It is especially relevant when the goal is to understand, resolve, or improve a procedure carried out in the professional world [43]. Considering the descriptive nature of the phenomenon, the extent of the literature, and the first-hand information available, the case study methodology has been used in this article.

Through this methodology, this article will provide an integrated model of work–life balance strategies to achieve the retention of valuable HR. Table 1 shows the stages of the case study. The study is carried out in a multinational company. The firm was founded in 1987 through a strategic agreement among four airlines in order to create a global distribution system (GDS) of airplane tickets. A GDS is a computerized network system operated by a company that enables automatic transactions among travel service providers (airlines, railway operators, car rental firms, hotels, travel agencies, etc.). The GDS integrates the services offered by the providers, gathering them in a platform, allowing the main clients of the GDS (travel agencies) to easily compare offers, prices, and services, and fulfil the needs and requirements of their own clients. In terms of market share, the analyzed firm is the largest GDS system, accounting for around 40% of travel agency bookings, and being used by around 90,000 travel agencies worldwide. 

There are several reasons behind the choice of this company. Firstly this is because they conceive human talent as a key strategic enabler, which ensures business sustainability and growth. As a technology based company, the attraction and retention of highly valuable human capital is especially relevant for the organization. Accordingly, the firm is strongly focused on developing talent attraction, recruitment, and retention policies. 

The goals achieved following these policies are related to increased productivity; lower absenteeism; a happier, less stressed workforce; improvements in employee health and well-being; a more positive perception of the company as an employer; greater employee loyalty, commitment, and motivation; and finally, a reduction in staff turnover and recruitment costs. Among their 18,673 employees around the world, there are more than 150 nationalities. Moreover, 90% of the workforce has a permanent contract. There is also a significant level of age diversity, with employees from different generations. In this regard, about 18% of the workforce is under 30 years old, while about 17% is over 50 years old. On the other hand, the vast majority (around 65%) is between 30 and 50 years old.

To achieve the objective of the study and in order to promote an open dialogue, evidence was collected through two semi-structured interviews conducted with three HR area managers from the three global headquarters. First was the Spanish HR manager, where the global strategy of the firm is developed by the departments of finance, strategy, legal, information technology, business processes, sales and purchasing, human resources, communications, and branding. In this country, 178 employees were hired in 2018. Second was the French HR manager from the research and development center, involving departments such as applied research, functional analysis, software development, and quality assurance. In 2018, more than 570 employees joined the company in France. Last was the German HR manager, where the data center of the firm is located and 77 workers were hired. The interview with the Spanish manager was conducted face-to-face, while for the French and German managers, the Webinar tool was used. The interview transcription was checked by the informants to confirm consistency with the ideas expressed verbally during the interviews. 

On the other hand, we conducted 54 open interviews with employees from different organizational levels and departments. Moreover, we maintained informal contact for five years in Spain. The diversity in the backgrounds of the informants, both in terms of their position in the organizational structure and the department, has led to different perspectives, enriching the analysis and the implications of the research.

The transcription of the information gathered was carried out using ATLAS.ti, a software frequently used by researchers to deal with qualitative data. The number of interviews and informants was not specified at the beginning of the research, and it was decided that a strategy of “theoretical saturation” would be followed to determine this. Interviews were conducted until it was perceived that informants reported similar and consistent information to support the validity and quality of the theoretical model [44]. 

As a complement to the interviews, we participated in meetings, communication plans, or events organized by the company, which promoted the values of the organization in HR management, such as commitment, development, integrity, and trust. Finally, to complete the principle of triangulation in the collection of information, we accessed internal documents. Thus, diverse files were consulted on the corporate intranet, in particular the handbook on the implementation of HR strategy 2014–2019, which was created and signed by the global human resources director of the group. 

## 4. Case Study Results: The Influence of the Work–life Balance Strategies on the Retention of Talented HR

**Figure 1:**
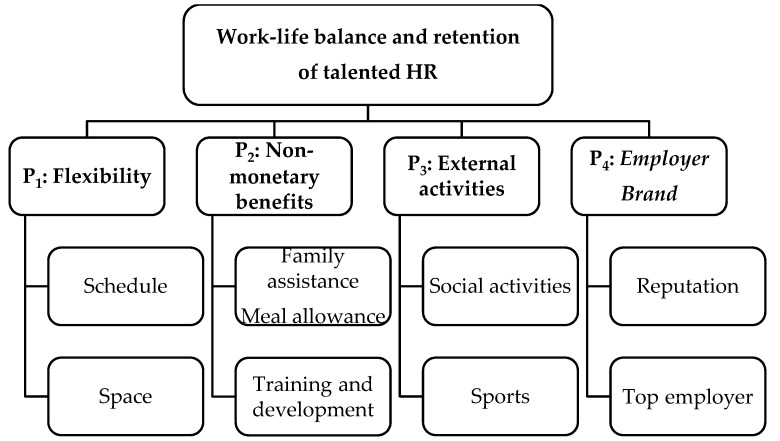
Strategies for work–life balance and retention of valuable HR.

Based on the literature of work–life balance strategies, the objective of this paper is to propose a model encompassing the most relevant factors for retaining valuable HR in an organization. As can be seen in Figure 1, the integrated model stems from previous research and has been analyzed together with the informants in the case study. Results from information gathering, transcription, and a summary are described below.

### 4.1. Schedule and Spatial Flexibility

Since time availability is one of the most valuable aspects for HR, flexible scheduling is one of the most common measures taken by firms. This consists of giving the workers the chance to decide the time they arrive and leave. The possibility of having flexibility in their schedules can help workers to improve their time management and to better juggle work time with social and family necessities. Another form of schedule flexibility can be a shorter workday, which allows for an uninterrupted shift. In this way, the employees are able to cut lunch-time, reduce down time in their workday, and thereby enjoy more time for themselves. 

The Spanish HR Spin manager described a frequent measure in the industry:

“*Within this type of flexibility there is the option of working according to annual hours. This practice drives talent retention, since it allows the HR to not only choose the time of arriving and leaving, but also to organize the hours that they work during the day and during the year, being able to work more intensive shifts that increase the number of their free days. This practice is common in project-oriented jobs*.”

The HR France manager highlighted unpaid leave or sabbatical periods:

“*The HR can sometimes find themselves in circumstances where they have to leave work in order to attend to personal necessities. With regards to preserving the talent of these HR, conceding time off for their personal matters can change their idea of abandoning the organization, and especially ensure create greater loyalty and commitment with the organization*.”

In addition, the manager also stressed that the company is determined to provide working parents with an environment that is better suited to people with family commitments:

“*We design specific arrangements for parents aiming at helping them with the day-to-day life of parents, to organize work in ways that adapt to their schedule, and to provide support from management and human resources. As an example, meetings are scheduled neither early in the morning nor late in the evening*.”

Depending on the sector of the company and the role of the worker, commuting to an office may be not necessary sometimes. The German HR manager explained:

“*The advance of technology, along with the tendency to work towards objectives instead of hours, allows for greater spatial flexibility. These measures can be critical for the satisfaction of employees, especially in big cities where the traffic or the distance between places causes HR to spend a lot of time in commuting. The most common measure is telecommuting. This measure allows the HR to develop their activity outside the office, from their homes or from a more convenient place. One last measure is that of e-learning. Developing online courses allows for the saving of commuting time and for making schedules more flexible in order to adapt to the needs of the employee*.”

Similarly, the Spanish HR manager highlighted the “smart work” program recently implemented in Madrid:

“*We offer the opportunity for the employees to perform part of their working activities outside the firm premises. The objective is to allow more flexibility to allow the employee to better engage with personal needs, without having to commit to a fixed teleworking regime*”.

Accordingly, our first proposition is the following:

**P_1_: Schedule and spatial flexibility has a positive effect on the retention of talented HR.**

### 4.2. Non-monetary Benefits

Non-monetary benefits, such as those of a social nature, are incentives designed to address a specific need and are often provided in a non-cash form. They are less widespread due to the high cost that they imply for the company. In the design of non-monetary benefits, it is important to keep in mind the priorities and the lives of the employees. Furthermore, the benefits must be aligned with the HR department policies and support the culture and values of the organization. 

With regard to the distribution of non-monetary benefits, the French HR manager argued:

“*Valuable HR look for companies with a conciliatory, open, meritocratic, diverse, and horizontal culture. In this regard, the corporate culture plays a key factor in retaining talent. If the objective is to reach a culture similar to this, these benefits should not be distributed according to the position of the employee in the organization; rather they should be applied to all the HR in order to achieve a structure that is as horizontal as possible*.”

Furthermore, non-monetary benefits have the greatest impact on employees, given that they may perceive that the company is not only interested in getting the maximum profit, but it also keeps in mind the individual needs of each worker beyond their job. This generates more commitment and a better retention of personnel. 

One of the most useful measures for HR is childcare assistance. However, only a few companies offer these policies. In addition to the high cost, some of the reasons why this kind of practice is not more widespread are problems related to space, an insufficient number of HR, problems with equality when it comes to compensating employees without children, or legal issues. In this regard, the French HR manager explained:

“*The company provides services and help parents mainly through nursery tickets, a childcare center, and a parenthood charter. These measures aim to help with the day-to-day life of parents, also by providing financial support. Moreover, we also offer training to managers to encourage them to take better account of their employees’ needs as parents. Finally, parents who decide to work part-time or take parental leave are not disadvantaged in respect to their career evolution*”.

Another kind of non-monetary benefit can be that of offering dining hall services or restaurant vouchers. Thanks to this, the HR do not have to dedicate free time after work to cooking and can dedicate more time to their personal matters. Accordingly, the French HR manager explained:

“*Employees appreciate meal allowances or facilities. Some innovative benefits are now being implemented in diverse parts of the company, including discounted fresh fruits offered to employees and healthy eating programs*”.

Career development is another concern for the workforce and for the firm’s success. In order to fulfil this need, companies can develop many kinds of training programs, both generic and specialized, which help HR to continue learning from their current roles and extend their knowledge within their company. Accordingly, the Spanish HR manager confirms this:

“*When employees are satisfied with job training, they are more likely to stay in the organization and have lower turnover intentions. In the same vein, this company places great importance on the continuous growth and development of our staff and have been steadily investing in training programs. The training programs offered are classifies into three categories: management and leadership programs aimed at newly appointed managers; soft competencies and behavior programs that focus on topics such as communication, negotiation, and time management; and knowledge-based programs that cover specific areas, such as software development, finance. and marketing*.”

In addition to general training programs, the German HR manager highlighted the importance of personalized training and education programs to retain the best talent:

“*We develop personal learning plans (PLPs) to ensure that employees are able to acquire the knowledge, understanding, and skills necessary to advance and develop their careers. These plans are adapted to employees’ needs and circumstances, and include diverse activities, such as on the job learning, coaching and mentoring, self-study, special projects, and feedback. These practices have resulted in better knowledge and skills transfer, open communication, preparation for change, and enhancement of leadership skills and management of teams*.”

Regarding the mentoring program, the Spanish HR manager reveals.

“*The third edition of Amadeus’ global mentoring program was in 2018, with the participation of 64 employees. The training consisted of behavioral fitness and positive leadership in collaboration with Madrid’s Business School University, which gave us exceptional feedback from attendees*.”

Based on the above, our second proposition is the following:

**P_2_: Non-monetary benefits have a positive effect on the retention of talented HR.**

### 4.3. External Activities

The ties that the worker creates with their colleagues and with the company itself can affect the decision to stay in an organization. It is essential to design, plan, and provide a good work environment. A good image will be that which demonstrates that the organization is accessible, professional, and focused on people. It is very interesting to do activities outside the office, such as internal contests to create new ideas in teams, activities to alleviate stress, as well as activities such as cooking workshops, learning foreign languages, or sports events. 

Regarding these options, the German HR manager stated:

“*Doing social or environmental activities can also improve the internal and external image of the company by demonstrating that it is committed to the society and by involving the HR with it. Another interesting idea is that of doing projects that are not related to the day-to-day work. These can be activities for the benefit of the company, such as decorating the office, or without benefit for the company, such as a photography contest. It is about offering activities that get the HR out of their routines and comfort zones, and increasing their creativity and interaction with people with whom they do not normally interact*.”

In addition to on-site activities, the Spanish HR manager tells us about the online platform called “live and learn”:

“*This initiative aims to drive collaboration, mutual trust, and respect among co-workers. It basically consists of home exchanges between Amadeus colleagues around the world to live as a local instead of visiting as a tourist, while saving money on accommodation costs. In addition, it also offers a linguistic exchange for employee’s children to learn or improve a foreign language by staying with a fellow colleague and their family for a few weeks*.”

Promoting and facilitating the practice of sports is a measure that is gaining popularity. More people are concerned about their health and about practicing sports as a hobby. The French HR manager spoke about the current method of work:

“*Technological progress has transformed jobs and it has caused job positions to require less physical effort, increasing sedentariness, favoring the increase of illnesses such as osteoporosis or diabetes*.”

The Spanish HR manager stressed the benefits for employees, and by extension for the firm, by promoting sport activities among the workers:

“*The practice of sports reduces the risk of illnesses, stress, and sick leave. Moreover, employees who practice sports have greater concentration and performance*.”

In the case of the French HR manager, they confirmed this:

“*In order to promote sports activities in France, we have reached agreements with facilities near the work offices, such as gyms or swimming pools. Furthermore, participation in races or sports leagues has also been promoted among employees*.”

Furthermore, the German HR manager highlighted the role of sport in the employee satisfaction with the organization:

“*The practice of group sports within the company can cause an improvement in teamwork and respect for colleagues, and strengthen relationships with other employees. A person who practices sports is exponentially happier and feels mentally better thanks to the generation of endorphins, which create pleasant sensations and positive thoughts. Moreover, a worker who is more positive and mentally rested is more creative and productive, and will have a better attitude*.”

Based on the above, our third proposition is the following:

**P_3_: External activities have a positive effect on the retention of talented HR.**

### 4.4. Employer Brand

Many companies are developing employer branding models as a method for attracting and retaining talent, especially amongst the youngest generations [35]. External branding represents an organization’s employer image and it is focused on potential employees to make them willing to join the company by projecting the image of being the best place to work. On the other hand, internal branding corresponds to an organization’s identity, aimed at retaining the current employees [45].

In this respect, the Spanish HR manager said: “The company has been rewarded several times with diverse “top employer awards”.

“*Employer brand is a combination of psychological, economic, and functional benefits provided to HR by their employer. This involves using marketing concepts in order to situate the company in the minds of the potential HR as a great place to work. The brand of the employer helps to create a positive image of the organization, sending the correct message to both the current and potential HR. The results of our 2018 employee engagement analysis, as well as the external recognition, suggest we are on the right path to achieving these goals. In this regard, the employee turnover in the company is 6%*.”

Regarding the importance of attracting talent, the French HR manager:

“*The careers of the HR who currently work in the company will reach an end one day. Generational change is inevitable. The future of the organization depends on the HR that we are currently recruiting, selecting, and socializing with. In this vein, we are strongly focused on social media and employer branding, and expanding our presence in top professional online boards, social media channels, and job platforms. We regularly publish our participation in events to promote what it is like to work with us, in order to increase the visibility of job opportunities to attract the best talent. Moreover, we are in continuous contact with top universities, recruitment fairs, and sponsoring, and we participate in hackathons*.”

Some of the concepts that identify HR with the brand are development, personal growth, success, strength, a good work environment, and a good social reputation. Therefore, the policies adopted by the HR department have to fit the image that the employer is creating and has to promote an environment of professional development, personal growth, and a positive atmosphere.

The German HR manager relates it to another key aspect in organizations:

“*It is a concept that has to be integrated into the organizational structure, to manage the work by objectives. What is more, the support of the managers and executives is important, and they should be the first to adopt and fulfil these policies. It is also about creating more horizontal teams, where concepts such as empowerment—where each person is responsible for their own work—are more and more important*.”

In an organization with a better employer brand, the value proposal is reflected in the actions of all the people in the business at all levels, stemming from a strong psychological contract between the company and its HR. According to this, our fourth proposition is the following:

**P_4_: The employer brand has a positive effect on the retention of talented HR.**

## 5. Discussion

Traditionally, organizations have focused on economic factors (earnings, profitability, ratios, etc.), however it has been demonstrated that the functioning of an organization depends on the attitude of its HR. HR has become a decisive factor for business success. It is essential that all the policies adopted are assumed as part of the culture of the organization and respected at all organizational levels, starting with HR managers. 

Globalization and competitiveness are critical issues making it more difficult for firms to recruit and retain the best talent. Moreover, social changes such as the aging of the population, the decrease in birth rates, or the new generation’s demands also affect talent policies. The effects of the previous elements make it necessary for firms to develop and apply talent management practices focused on the systematic attraction, identification, development, engagement, retention, and deployment of talent. These practices will have a positive impact on human resource outcomes, such as job satisfaction, motivation, and commitment, and also on the firm performance.

In this context, work–life balance is gaining relevance in the current business context, as companies are facing greater management difficulties, which in many situations entail the loss of valuable HR. There is frequently a gap between the factors demanded by the HR in order to satisfy their needs and motivations and the measures implemented by the organizations. 

Accordingly, the aim of the research is to understand if investing time and resources in managing work–life balance helps to attract and retain valuable HR. With the aim of responding to the research question, three objectives have been accomplished. Firstly, we analyze diverse society changes driving working balance policies. Second, the relation between work–life balance policies and talent management is addressed. Finally, through a case study we propose a roadmap for HR managers, consisting of an integrated model of key work–life balance policies to retain talented employees.

In Table 2, the propositions suggested in the model are summarized, along with the HR practices enacted by the organization related to work–life balance strategies and the results of valuable HR retention. Along with the reflections made by the three HR managers in the company, our study also included 54 open interviews with people from different organizational levels and different departments, along with informal contact with them during the five years. Their words and experiences support the propositions previously presented and the “work–life balance practices” and results of HR retention” in Table 2.

## 6. Conclusions

From our work, two kinds of contributions are derived. For the scientific community, the article offers a model of actions to be able to retain HR through work–life balance strategies, integrating previous theoretical contributions from specialized authors, and corroborating their research with first-hand information from a multinational company that is a leader in its sector. Therefore, the results of the article build up a complete and detailed framework, including the most relevant work–life balance policies, along with the direct positive impact we can expect from them. This could be the basis of future studies carried out under different external (e.g., country or industry influence) or internal (e.g., organizational structure, culture, or leadership style) influences that may empower of modify the effects of such policies.

Moreover, the study offers a series of practical implications. A roadmap has been drawn up that can serve as a reference for the different managers involved. When a firm is talent-based, managers cannot ignore the need to put some, if not the majority, of these policies into practice, so that the firm is attractive in the talent market, which will in turn be needed to keep its competitive edge. This work could be a basic catalogue of actions to be adapted and implemented. In this way, each manager will know the most relevant HR factors that must be managed and the actions that can be carried out to achieve success in the process.

The management of HR is an increasingly relevant theme of critical importance for the future of organizations. Therefore, it is necessary to develop this research stream. One initial line of future research would be using quantitative evidence in order to complement the results of the case study. A second line of future research would require analyzing whether there are significant differences in the impact on capturing or retaining valuable HR among industries and drawing conclusions for each of them. A third line of research could focus on the effects of the combination of different compensation methods, considering not just work–life balance, but its potential combined effects with some other approaches, such as equity participation or inside debt. Finally, a fourth line of research could be a comparative study between HR in different stages of their development or professional careers; for example, valuable HR who have recently graduated from university and are in the first stage of their professional careers, compared to mid-level executives in the middle of their careers, or to executives in high-level positions in the organizational structure. 

## Figures and Tables

**Table 1:** Stages of the case study.

**Profile** **Review of Literature (Web of Science and Scopus)**
**Sample selection** Technology and tourism industry firm Spain, France, and Germany strategic business units from a multinational company
**Units of analysis** Human resources (HR) department manager Different informants who participated in the implementation of the work–life balance strategies
**Information gathering** Semi-structured interviews: 2018 (HR managers) Open interviews: 2017–2018 (employees) Internal documents: 2014–2018 Direct observation: 2014–2018 (Principle of triangulation)
**Information transcription** ATLAS.ti (qualitative data analysis) Data records and classification: (1) internal documents, (2) interviews, (3) field notes, and (4) direct observation
**Results and conclusions** Conformity with the analysis results Conclusions, along with literature and professional implications

**Table 2:** Propositions, practices, and results regarding work–life balance management and their implications in the retention of valuable HR.

Why does Investing Time and Resources in Managing Work–life Balance Help to Attract and Retain Valuable HR?		
Propositions	Work–life Balance Practices	Results of HR Retention
P1: Schedule and spatial flexibility	Short workday Annual hours Technological advances	Social needs Rest periods Commuting
P2: Non-monetary benefits	Daycare assistance Meal allowances Training programs	Family needs Time saving Career development
P3: External activities	Team activities Internal contests Sports	Integration Stress management Health and well-being
P4: Employer brand	Social marketing Work environment Empowerment	Positive image Young talent Horizontal teams
